# Supplementary material for: Replicating RNA platform enables rapid response to the SARS-CoV-2 Omicron variant and elicits enhanced protection in naïve hamsters compared to ancestral vaccine
Source: eBioMedicine. 2022 Aug 4;83:104196. doi: 10.1016/j.ebiom.2022.104196 (PMC9349033; doi:10.1016/j.ebiom.2022.104196)
Supplement: Supplementary file 2 [file mmc2.pdf]

Weight Loss

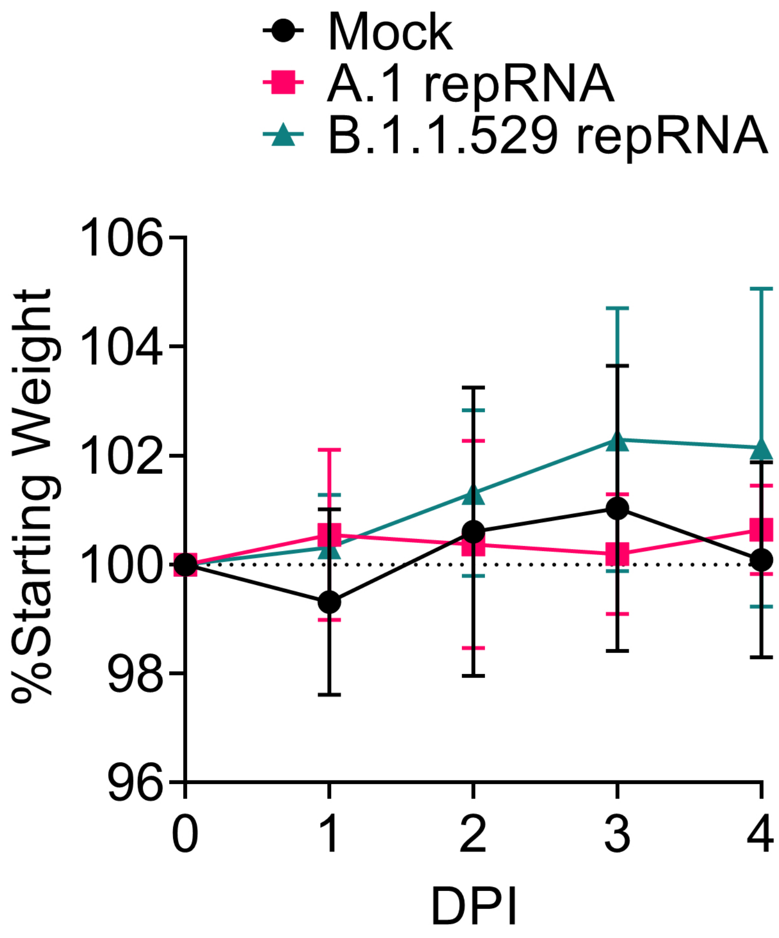

Supplemental Figure 1: Weight loss in B.1.1.529-infected hamsters. Hamsters vaccinated with indicated repRNAvaccine were challenged with 1000 TCID50 of B.1.1.529 via the IN route four weeks after vaccination. Hamsters were weighed daily. Data presented as mean plus standard deviation. N = 6 per group.
