# Supplementary material for: Replicating RNA platform enables rapid response to the SARS-CoV-2 Omicron variant and elicits enhanced protection in naïve hamsters compared to ancestral vaccine
Source: eBioMedicine. 2022 Aug 4;83:104196. doi: 10.1016/j.ebiom.2022.104196 (PMC9349033; doi:10.1016/j.ebiom.2022.104196)
Supplement: Supplementary file 3 [file mmc3.pdf]

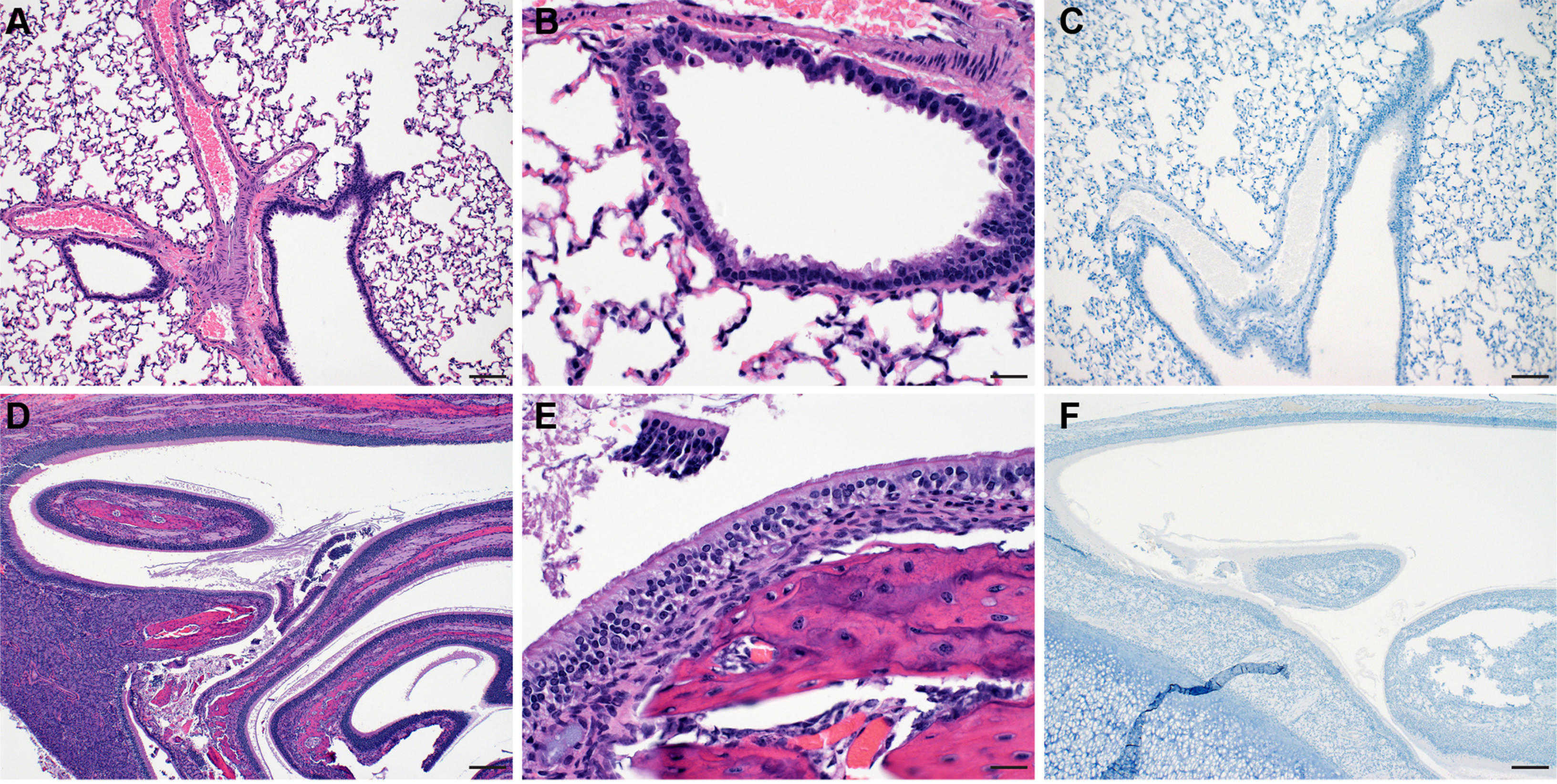

Supplemental Figure 2: Tissue sections from historical mock-infected controls. a-b H&E sections of lung tissue. c IHC for SARS-CoV-2 antigen in lung tissue. d – e H&E sections of nasal turbinates. f IHC for SARS-CoV-2 antigen in nasal turbinates.
